# Recognition of Cattle's Feeding Behaviors Using Noseband Pressure Sensor With Machine Learning

**DOI:** 10.3389/fvets.2022.822621

**Published:** 2022-05-25

**Authors:** Guipeng Chen, Cong Li, Yang Guo, Hang Shu, Zhen Cao, Beibei Xu

**Affiliations:** ^1^Agricultural Economics and Information Institute, Jiangxi Academy of Agriculture Sciences, Nanchang, China; ^2^AgroBioChem, Precision Livestock and Nutrition Unit, Gembloux Agro-Bio Tech, University of Liège, Gembloux, Belgium; ^3^Information Technology Group, Wageningen University and Research, Wageningen, Netherlands; ^4^Agricultural Information Institute, Chinese Academy of Agriculture Sciences, Beijing, China

**Keywords:** noseband pressure sensor, machine learning, XGB, behavior classification, feeding behaviors

## Abstract

Automatic monitoring of feeding behavior especially rumination and eating in cattle is important to keep track of animal health and growth condition and disease warnings. The noseband pressure sensor is not only able to accurately sense the pressure change of the cattle's jaw movements, which can directly reflect the cattle's chewing behavior, but also has strong resistance to interference. However, it is difficult to keep the same initial pressure while wearing the pressure sensor, and this will pose a challenge to process the feeding behavior data. This article proposed a machine learning approach aiming at eliminating the influence of initial pressure on the identification of rumination and eating behaviors. The method mainly used the local slope to obtain the local data variation and combined Fast Fourier Transform (FFT) to extract the frequency-domain features. Extreme Gradient Boosting Algorithm (XGB) was performed to classify the features of rumination and eating behaviors. Experimental results showed that the local slope in combination with frequency-domain features achieved an F1 score of 0.96, and recognition accuracy of 0.966 in both rumination and eating behaviors. Combined with the commonly used data processing algorithms and time-domain feature extraction method, the proposed approach improved the behavior recognition accuracy. This work will contribute to the standardized application and promotion of the noseband pressure sensors.

## Introduction

Precision livestock farming (PLF) is a research field involving multiple disciplines such as the Internet of Things (IoT) and artificial intelligence (AI). Through the continuous real-time monitoring of individual livestock's health and growth condition, not only the animal welfare but also the production and quality can be improved further (1, 2). Feeding behavior is one of the key indicators in cattle to measure growth and diseases. Accurate analysis of feeding behavior can help evaluate the feed intake and growth rate, which could be used to provide a reference for cattle breeding (3). In particular, rumination and eating are the most direct and effective feeding behavior characteristics to confirm the cattle's health status (4). Therefore, automatic and accurate monitoring of rumination and eating in cattle is of great significance to building precision livestock.

In traditional livestock farming, direct observation is one of the most widely used methods (5). Stopwatch, counter, telescope, and other tools are commonly used to track cattle for continuous recording of their eating, rumination, resting, and wandering behaviors. Unfortunately, these methods are both time and labor-consuming, especially for the grazing sector (6). With the development of information technologies, sensor technology has been applied to monitor cattle behaviors to achieve automatic and continuous detection, which is more efficient and less intrusive than the traditional manual monitoring method (7). Due to the favorable stability and endurance, wireless sensors can provide long-term monitoring, and therefore, are promising in grazing pastures (8). The sensors consist of sound sensors (9, 10), acceleration sensors (11, 12), and pressure sensors (13, 14). The sound sensors are mainly used to detect the chewing behaviors of cattle such as chewing, biting, and chew-bite through the sound produced during chewing (15, 16). For example, Chelotti et al. (17) proposed an online bottom-up foraging activity recognizer algorithm incorporating multilayer perceptron (MLP) along with a decision tree and achieved the F1 scores of 82.2% (grazing) and 74.3% (rumination) in the 5 min detection window size. Although the sound sensors have good performance for monitoring chewing behavior in the ideal environment, they are susceptible to being affected by noise in complex farms (18). The monitoring method based on acceleration sensors is to fix the devices on the head, mandible, ear, neck, or other parts of cattle and then identify the feeding behaviors by distinguishing the movements and postures of the acceleration (19, 20). For instance, Smith et al. (7) used the triaxial acceleration to collect cattle motion data signals and the one-VS-all classification framework was proposed to recognize grazing, walking, ruminating, resting, and other behaviors. In the 30 s window size condition, this method achieved the F1 score of 0.98 (grazing) and 0.86 (ruminating). The acceleration sensors are always affected by the semblable acceleration signals and different behaviors may have semblable signal features, it is difficult to judge the behaviors for acceleration-based models, especially in practical scenarios (21). Given that sound sensors and acceleration sensors have limitations in the task of behavior recognition, some studies have improved the classification performance by combining multiple sensors (22, 23).

The environment of pasture is more complex than that inside farmhouses, and thus, it is more challenging to monitor the feeding behavior. Compared with sound and acceleration sensors, noseband pressure sensors can directly sense the pressure change of cattle's jaw movements and reflect the chewing behavior, which is a direct and effective way to monitor real-time feeding behavior (24). Since the pressure signal of sensors and cattle's chewing behavior have a very high correlation (25). Rutter (26) proposed the program (called Graze) that was used to apply the amplitude and frequency of pressure data to conduct the behavior identification of eating and rumination and obtained 91% identification accuracy. However, Graze was used in conjunction with the IGER Behavior Recorder, and the recorder interfered with the animals. Zehner et al. (27) developed and validated a novel scientific monitoring device for automated measurement of ruminating and eating behavior in stable-fed cows to provide research with a measuring instrument, and published two software versions of RumiWatch to identify cattle foraging behavior, and the average recognition accuracy of the two software versions was 88.86 and 85.31% under the condition of 1 min windows. Subsequently, several versions of RumiWatch were released, and the prediction accuracy of ruminant behavior and foraging behavior in the 1 min window were both higher than 90% (28, 29). Shen et al. (30) applied the pressure sensor to count rumination bouts, duration of rumination, and the number of cuds, respectively, and obtained an accuracy of 100, 94.2, and 94.45%. They proposed to use the SD and spectrum characteristics of pressure data for rumination behavior recognition and achieved an accuracy of 94.2% under the condition of 51.2 s time resolution.

The monitoring of feeding behavior could help predict herbage demand (31). Consequently, to monitor the cattle's health more effectively and improve animal welfare, the feeding behavior recognition algorithm based on pressure sensors needs more progress for future potential applications (32). However, in previous studies, the pressure sensor-based feeding behavior recognition algorithm is built on the peak rates and the peak intervals of cattle chewing data. The peak is detected by the threshold, which is very sensitive and easily affected by the pressure values. Due to the difference in the size of cattle head, to obtain the initial state (trough of the pressure value) of the chewing process, it is necessary to ensure that the jaw is fully occluding, and the flexible band is at the same stretch degree. To achieve this, there is a need to observe the jaw movement and the pressure value of the equipment at the same time and then adjust the equipment accordingly, which is quite laborious. Furthermore, the maximum pressure of complete opening is still out of control. Moreover, the classification of feeding behavior is based on general algorithms without animal-specific learning data, and thus the average recognition accuracy is low.

Machine learning (ML) methods have been widely considered in feeding behavior recognition, namely, support vector machine (SVM), random forest (RF), and extreme gradient boosting (XGBoost) (33). Dutta et al. (34) adopted supervised machine learning techniques for cattle behavioral classification with a 3-axis accelerometer and magnetometer, and the highest average correct classification accuracy of 96% was achieved using the bagging ensemble classification with Tree learner. Fogarty et al. (35) explored four ML algorithms (CART, SVM, LDA, and QDA) for sheep behavior classification with ear-borne accelerometers, and the accuracy for each ethogram was over 75%. Riaboff et al. (36) developed a prediction method for feeding behavior and posture using accelerometer data based on the XGB algorithm and also presented a variety of machine learning algorithms for comparisons. Balasso et al. (3) developed a model to identify posture and behavior from the data collected from a triaxial accelerometer located on the left flank of dairy cows, and four algorithms (RF, KNN, SVM, and XGB) were tested and the XGB model showed the best accuracy. Dutta et al. (37) developed and deployed a neck-mounted intelligent IoT device for cattle monitoring using the XGBoost classifier, which achieved an overall classification accuracy of 97%. Although XGB algorithms achieved good performance in accelerometer data analysis, the XGB algorithms with noseband pressure sensors have never been applied to the classification of livestock behavior. Therefore, there is a need to assess the application of the XGB algorithm to recognize livestock behaviors with noseband pressure data.

To simplify the standardizing of the wearing of the nasal band pressure sensor and improve feeding behavior recognition, this article aims to explore a data processing approach in combination with a machine learning model to eliminate the impact of different ranges of noseband pressure values and facilitate the behaviors recognition.

## Materials and Methods

### Experimental Equipment

**Figure 1:**
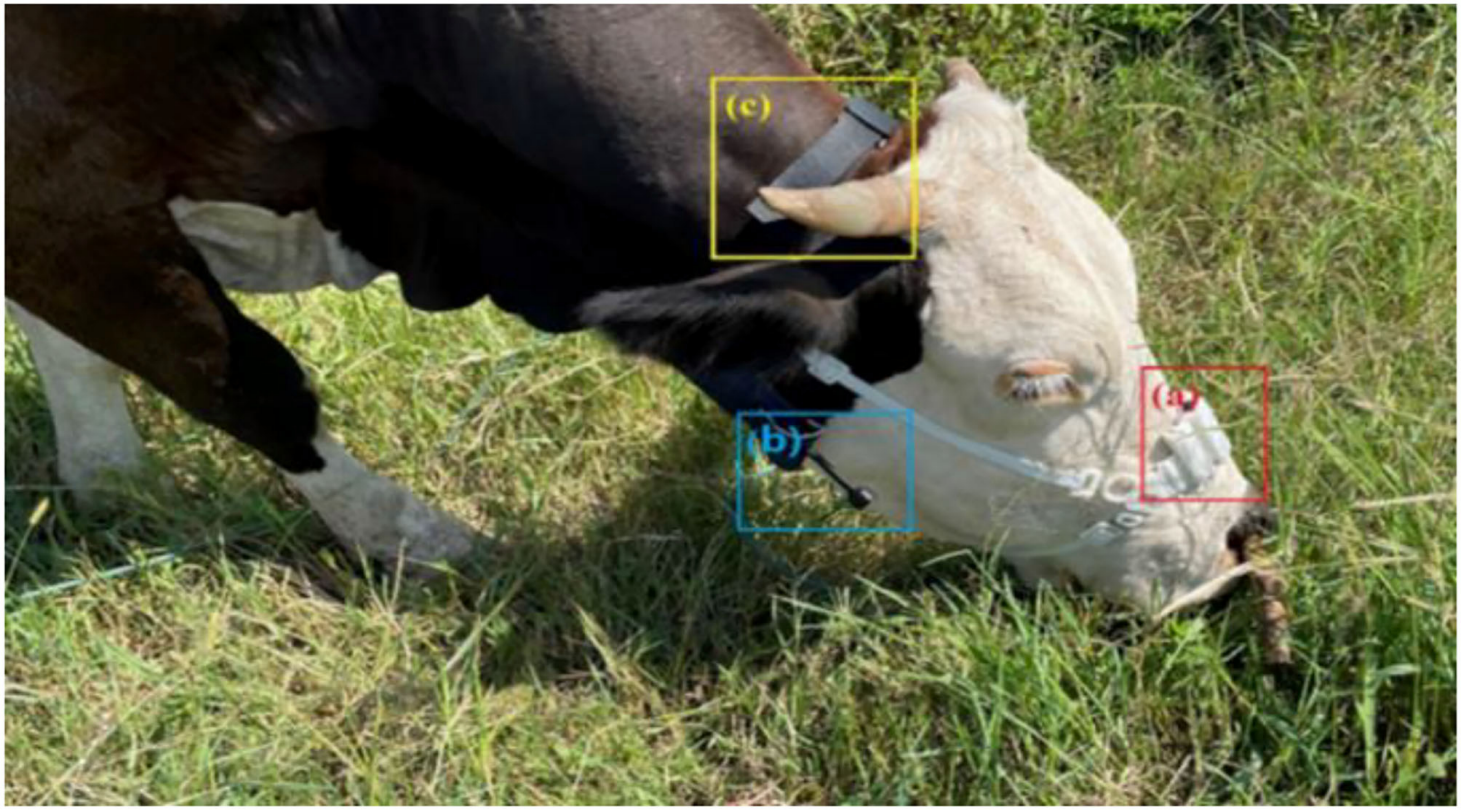
Components of the wearable integrated data acquisition device. (a) Masticatory pressure collecting device, (b) miniature camera, and (c) adjustable wearable collar.

**Figure 2:**
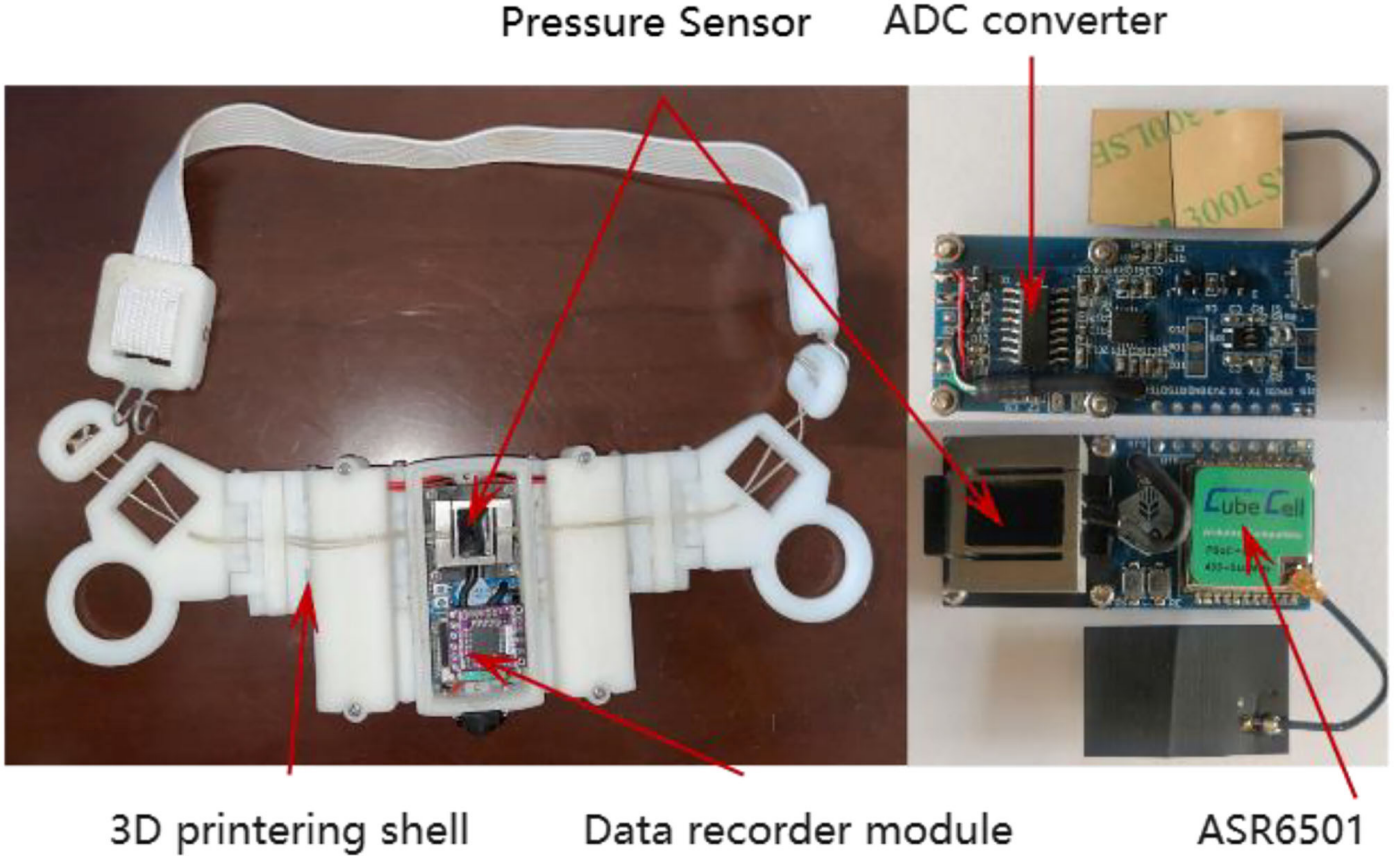
Masticatory pressure collecting device.

This research developed a set of comprehensive data acquisition equipment, which could collect masticatory pressure data and record behavior data in videos at the same time. The equipment consisted of the masticatory pressure collecting device, the miniature camera (Gold touch U21), and the adjustable wearable collar, as shown in Figure 1. The Masticatory pressure collection device included a pressure sensor (0–2,000.000 g), an HX711 ADC converter (sampling frequency 50 Hz), an SoC chip ASR6501 integrated with LoRa, a 16 GB data recorder module, a 3D printing shell, and two lithium batteries (3.7 V, 500 mAh) in Figure 2. Three stretchable cords attached the device to the head of a cow. Since it is very difficult to track the chewing behavior of cattle manually under natural grazing conditions, a micro camera (64 GB SD card), and mobile power supply (5 V, 10,000 mAh) were used to construct a wearable video monitoring system for chewing behavior by 3D printer, to ensure that the whole process of cattle chewing behavior was collected for a long time. The video monitoring system can maintain 24 h of uninterrupted power supply, in which the working currents of data acquisition equipment and miniature cameras are 26 and 230 mA. The mobile power not only supplies power to the miniature camera but also charges the masticatory pressure collecting device. Changing the mobile power supply once a day can maintain the continuous collection of pressure data and video data without dismantling equipment.

### Data Collection and Preparation

This research was conducted in the Gao'an base of the Jiangxi Academy of Agricultural Sciences. Three Simmental × Chinese Yellow crossbred cattle, each weighing about 650 kg on average, were used for grazing in the experimental area. Before binding to the cattle, the noseband pressure sensor device was turned on and automatically calibrated for an initial pressure value of 0 g for all devices. At the same time, the breeders helped make the cattle gentle, so that the noseband pressure sensor device and micro-camera could be installed. In addition, the equipment adaptability test on all three cows lasted for 2 days. The formal experiment period lasted for 5 days, from 8 a.m. to 5 p.m. every day. A total of 135 h of masticatory pressure data and the corresponding video monitoring data were collected. Based on the monitoring data of masticatory pressure collected by the micro-camera, the data of masticatory pressure were manually marked. As shown in Table 1, according to the suggestions from animal husbandry experts, the marking rules of cattle feeding behavior were summarized. Then, the annotated pressure data was saved in CSV files, where each line included the recording time, pressure value, and the label. It is worth noting that the noseband pressure sensor device was set up to synchronize with the micro-camera, ensuring that the behavior of the video annotation was corresponding to the original pressure data.

**Table 1:** The labeling rules of the classification behavior description.

**Behavior**	**Description**
Ruminating	Regurgitation, remastication with steady frequency, and swallowing
Eating	Bowing the head, moving grass from the pasture with the tongue into the mouth, chewing
Other	All remaining behaviors

### Data Preprocessing

The scope of original pressure data of different individuals varies greatly, and such scale difference has a great influence on the data modeling (38). In this section, data preprocessing was designed to obliterate the scale difference which was produced by the original pressure data. In the mastication data collected from cattle, different initial pressure values were generated after wearing the noseband pressure sensor due to the size difference of the cattle head. This initial pressure value is a relatively stable constant throughout the wearing process of the device. There are two ways to eliminate this constant, one is to extract local changes in the data, and the other is signal filtering. In this study, first-order difference and local slope were used to extract local variation of data. At the same time, the high-pass filter was used to filter out unstable initial variables. Furthermore, the control method exerted in this article means the original pressure data without any processing.

#### First-Order Difference

First-order difference (FOD) is the simplest way to extract the continuous numerical changes for a given sequence. More specifically, each term in the original sequence is subtracted from the next. After the above operation, the number of arrays is reduced by 1. Specific operations are as follows:


(1) $$\begin{array}{r} d_{t}=x_{t}-x_{t-1} \end{array}$$

#### Local Slope

Local slope (LS) is computed using linear regression over the spectral amplitude values (39). It can obtain the variation trend of local signals in samples. The algorithm can eradicate the initial pressure, and this is because the slope of the local signal is solely determined by the trend of signal change. The process of the local slope extraction is to extract all slope features of the data sample according to the sliding window with 11 sampling points and one stride. First, calculating the local slope requires an auxiliary arithmetic array, as shown below:


(2) $$\begin{array}{r} C=\left(1,2,3,\ldots ,9,10,11\right) \end{array}$$

To make the local slope in agreement with the current window, the least square solution is applied:


(3) $${\hat{s}}_{t}=\frac{N\sum_{i=1}^{N}c_{i}x_{i}^{*}-\sum_{i=1}^{N}c_{i}\sum_{i=1}^{N}x_{i}^{*}}{N\sum_{i=1}^{N}c_{i}^{2}-{\left(\sum_{i=1}^{N}c_{i}\right)}^{2}},N=11$$

**Figure 3:**
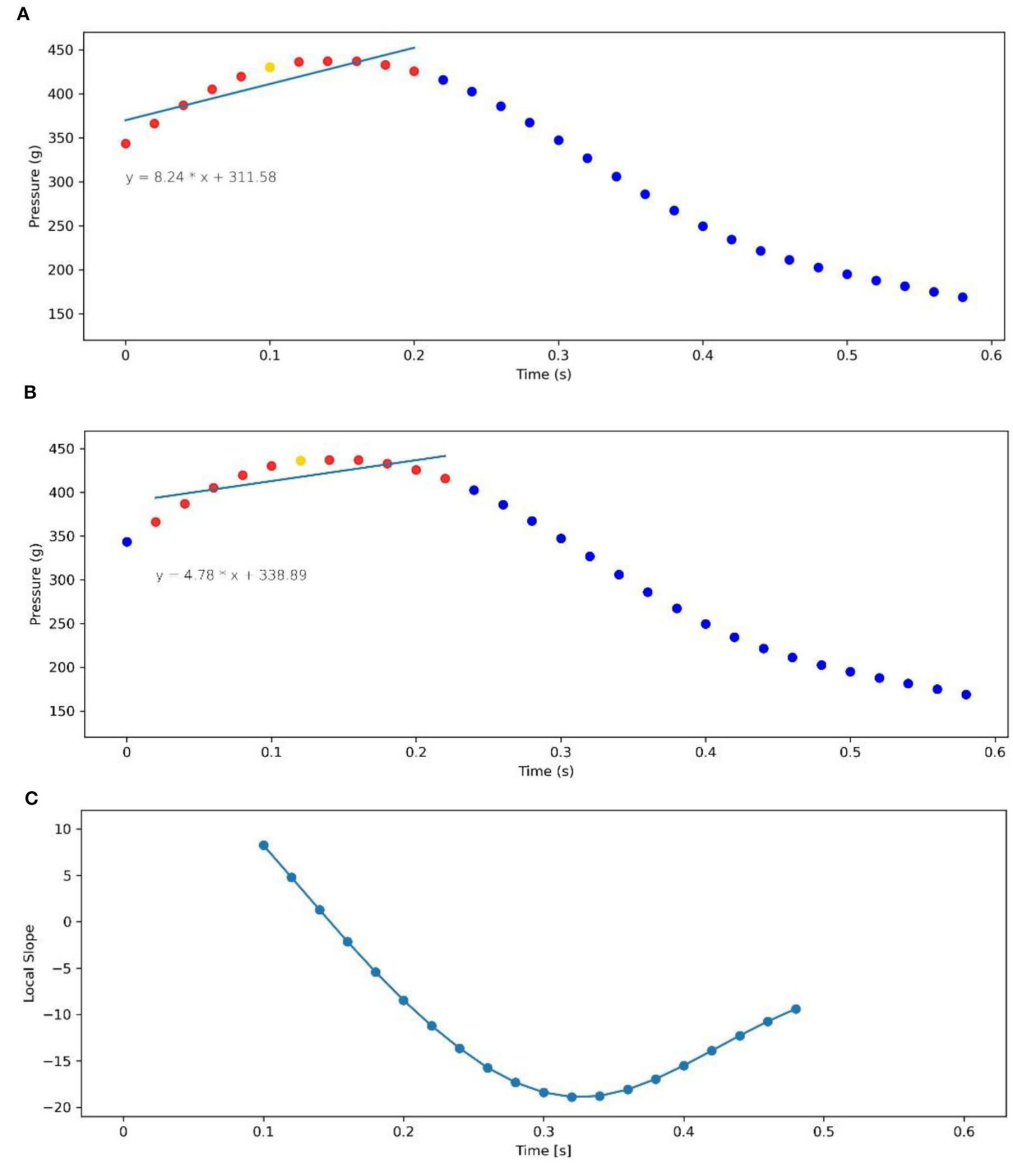
Illustration of the preprocessing operation of partial slope extraction. **(A)** Aims to extract the slope of the position of 0.1 s and the corresponding least-squares fitting line. **(B)** Intends to extract the slope of the position of 0.12 s and the corresponding fitting line. **(C)** It is the local slope after data preprocessing, each point of the local slope corresponding to the original pressure data of local trends. A total of 20 local slopes are extracted excluding the auxiliary calculation points.

$x_{i}^{*}$ is the sample point in each sliding window. The sliding window exhibits the local slope sample of the whole data sample. Figure 3 shows the process diagram of the partial slope extraction preprocessing method in the flow chart. After this process, 20 local slopes are extracted from 0.1 to 0.48 s corresponding to the raw pressure data.

#### High-Pass Butterworth Filter

High-pass Butterworth filter (HBF) is used to remove the frequency region in the waveform whose signal frequency is lower than the cutoff frequency, including the uncertain initial pressure value (40). Specifically, the original signal is processed with a high-pass Butterworth filter with a sixth-order cut-off frequency of 0.3 Hz. The output data points are the same as the input.

### Feature Extraction

Features were extracted from two aspects of the time and frequency domain. The frequency-domain feature (FDF) is established on the fast Fourier transform principle (41), as the range of unilateral frequency domain (not normalized). A sequence array of length 250 was extracted as an FDF, exhibiting both the range of intensity in each sampling frequency (0–25 Hz, resolution: 0.1 Hz) and serving as a distributed representation of the preprocessed data. The time-domain statistical feature (TDSF) included position parameters like average, variance, SD, maximum, minimum, range, median, first quartile, third quartile, interquartile range, root mean square, movement variation, skewness, and kurtosis. Skewness and kurtosis extracted in the time domain (34) were computed and the maximum of the pairwise correlations between each axis was considered. The time-domain feature extraction referenced the Riaboff method in the feature extraction process of acceleration sensor data for cattle behavior classification (42). Since the main frequency signal in the process of eating (around 1.3 Hz), ruminating (around 0.7 Hz) has a significant difference in the frequency domain, and time features were widely used in cattle behavior classification. Therefore, this article used FDF and TDSF for the data preprocessing.

### Model Training

In this section, the predictions of cow behaviors were carried out using a single classifier to compare the results. Extreme Boosting Algorithm (XGB) is a supervised ensemble learning algorithm (33). Its principle is to output the prediction probability of the corresponding category based on the training samples with multiple features and to find the category that is most probable to predict the kind of cow feeding behavior. XGB serves as an efficient way to mine latent patterns in data. This framework contains multiple hyperparameters, commonly used as follows:

- n_estimators: number of gradient boosted trees.
- max_depth: maximum tree depth for base learners.
- learning_rate: boosting learning rate.
- gamma: minimum loss reduction required to make a further partition
- on a leaf node of the tree.
- subsample: subsample ratio of the training instance.
- min_child_weight: The minimum sum of the required instance weights in a child.

The complexity of the model is directly influenced by the number of estimators and the max depth of the tree, which means that the greater the number of the trees are, the deeper the trees' depth is, and the more complex the model is. To compare the model capacity in this article, the same hyperparameters were used in all of the classifiers as seen in Table 2.

**Table 2:** The setting of hyperparameters of XGB algorithm.

**Setting**	**Parameters**
Global setting	n_estimator = 100 max_depth = 6 learning_rate = 0.4 gamma = 0.1 subsample = 0.8 min_child_weight = 1

### Model Validation

The performance of eight combined models was tested under the same parameters to find the optimal algorithm for data preprocessing and feature extraction. One way to assess model performance objectively is to utilize the cross-validation technique (43). It is used to prevent overfitting in prediction models, and protect against overfitting in a predictive model, particularly in a case where the amount of data may be limited. The major step of cross-validation is to divide all the data into five subsets, each of equal size. Four subsets were used for training, and the left one was used for testing. To ensure that no data were omitted in the prediction, the procedure was repeated 5 times and the confusion matrix was obtained for each time, which was then computed to form a total confusion eventually (44).

Accuracy, that is the proportion of successfully classified samples in total samples, serves as the standard for assessing the overall performance of classifiers. On the other hand, accuracy also reflects the ratio of correctly classified samples to total samples. To evaluate the prediction of true positive (TP), false positive (FP), and false negative (FN) in each scenario, precision, recall, and F1 score were also computed. The calculation of evaluation indicators is as follows:


(4) $$\begin{array}{r} precision=\frac{TP}{TP+FP} \end{array}$$

(5) $$\begin{array}{r} recall=\frac{TP}{TP+FN} \end{array}$$

(6) $$\begin{array}{r} F1=2*\frac{\left(precision*recall\right)}{\left(precision+recall\right)} \end{array}$$

## Results

### Multi-Window Performance Results

Noseband pressure sensors have been used to classify feeding behaviors, but classification accuracy and computational load are influenced by signal segmentation. Therefore, this article considers the accuracy and execution time as standard evaluation criteria to evaluate variable window size.

Table 3 shows the results of 1,000 samples with different window sizes (5, 10, 15, 20, and 30 s) and different method combinations. As can be seen, the accuracy achieved higher with the window size increased, but with the longer execution cost. The window sizes of 5 s led to significantly lower accuracy than with other window sizes but the execute time was also the fastest with 11.592 s. Despite the higher accuracy the larger windows size performed, considering the processing speed for potential use, this article selected the window size of 10 s for the data segmentation to balance the accuracy (over 95%) and execution cost. The dataset in this work contains a total of 47,482 samples, namely, 14,321 for rumination, 17,978 for eating, and 15,183 for other behaviors. The entire pressure dataset construction process did not use the data overlapping technique, and each sample was used only once in the original annotation data.

**Table 3:** The effect of window size on the accuracy (mean and standard deviation) and the executing time.

**Time windows**	**Accuracy**	**Execute time (1 k sample)**
5 s	0.944 ± 0.012	11.592 s
10 s	0.966 ± 0.010	21.440 s
15 s	0.973 ± 0.010	31.621 s
20 s	0.975 ± 0.014	41.717 s
30 s	0.981 ± 0.010	63.327 s

### Data Preprocessing Results

**Figure 4:**
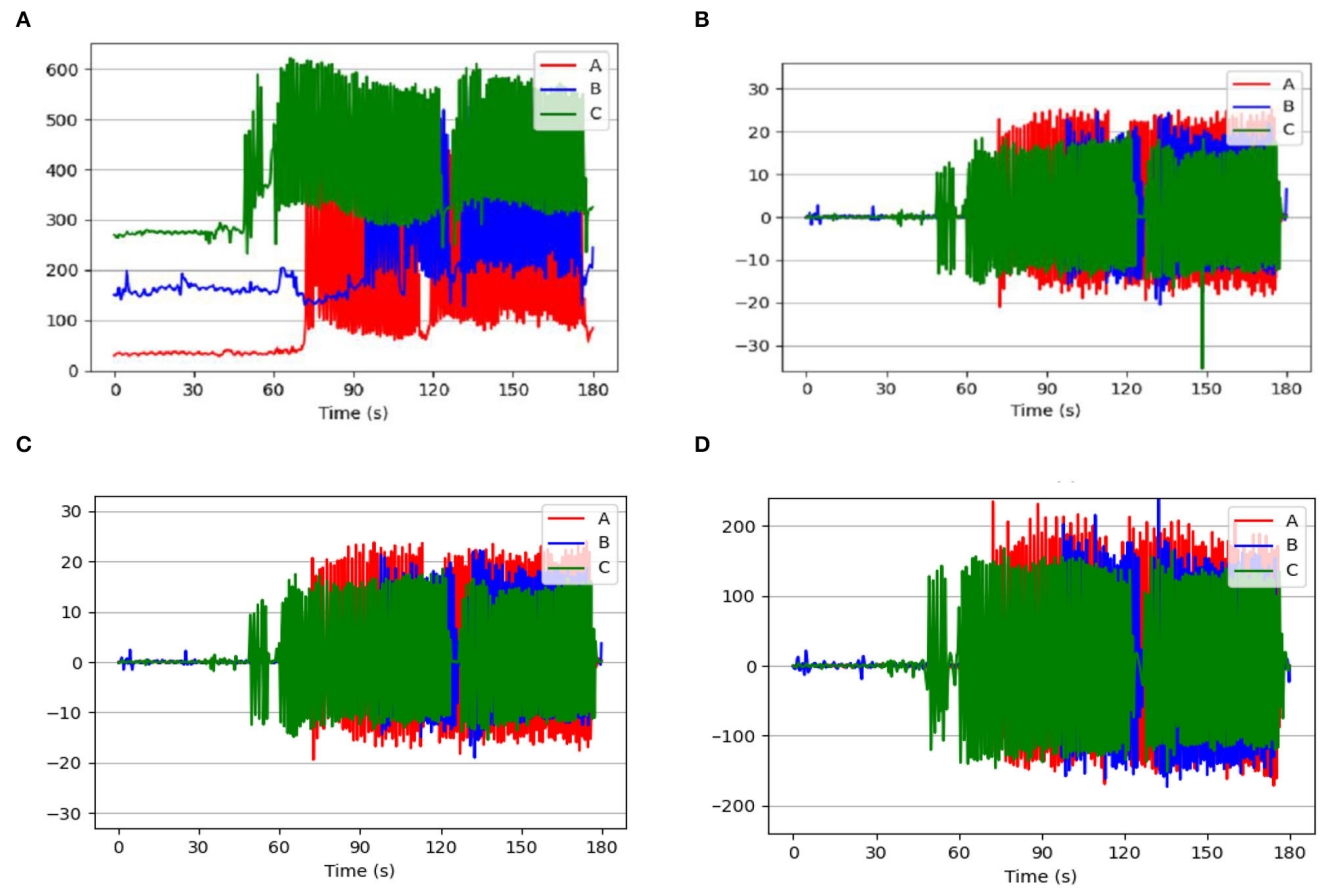
Graph of data pretreatment algorithm. Sample A, sample B, and sample C come from three different cattle. **(A)** Raw pressure data. **(B)** Data processed by the first-order difference method (FOD). **(C)** The local slope (LS) of raw data. **(D)** Data processed after the high-pass filter (HPF).

During the experiment, although this work was able to initialize the zero-calibration process when the device was not worn and placed horizontally and freely, it was unable to guarantee the same initial pressure value during the wearing process. The difference in initial pressure values of cows wearing the pressure sensor reflected the overall data on the feeding behavior (eating, ruminating, and others) of the three cows. Figure 4A shows the pressure data of three cows' ruminating behavior during the 180 s. Based on the three data preprocessing methods discussed in this work, the data processed by FOD, LS, and HBF are presented in Figures 4B–D. The minimum values of the original pressure waveforms obtained from the three cows were 28.550, 128.240, and 232.320 g, and the corresponding average waveforms values were 145.549, 235.616, and 385.515 g, respectively, as shown in Table 4. Accordingly, the cows with pressure sensors have different initial pressure values. However, the mean values for all three cattle processed by FOD, LS, and HBF were basically close to 0, which means the data preprocessing methods contribute to eliminating the influence of the initial pressure value. In particular, FOD is a kind of algorithm that uses two sampling points to extract the slope, and it has a small amount of calculation and range of variation and also is sensitive to mechanical noise. By comparison, LS adopts 11 sampling points to extract the slope, which takes into account a large range of changing trends, but costs a large amount of calculation. This method can effectively suppress noise and the pressure data are closer to the changing trend of the original. As shown in Figure 4, the FOD algorithm is sensitive to mechanical noise and produces sudden changes during 120–150 s, but the LS algorithm can eliminate the abrupt effects of sensors and behaviors and thus improve the recognition accuracy.

**Table 4:** Numerical statistical comparison of ruminant waveform data preprocessing.

**Method**	**Cattle**	**Min**	**Max**	**Mean**
Raw	A	28.550	450.660	145.549
	B	128.240	569.280	235.616
	C	232.320	622.585	385.515
FOD	A	−21.020	25.250	0.006
	B	−20.510	24.680	0.010
	C	−35.420	20.070	0.006
LS	A	−19.396	23.958	0.006
	B	−19.003	22.197	0.008
	C	−15.455	18.529	0.006
HPF	A	−171.611	234.801	−0.004
	B	−173.212	249.433	−0.025
	C	−151.353	168.116	−0.009

### Overall Performance of the Model

**Figure 5:**
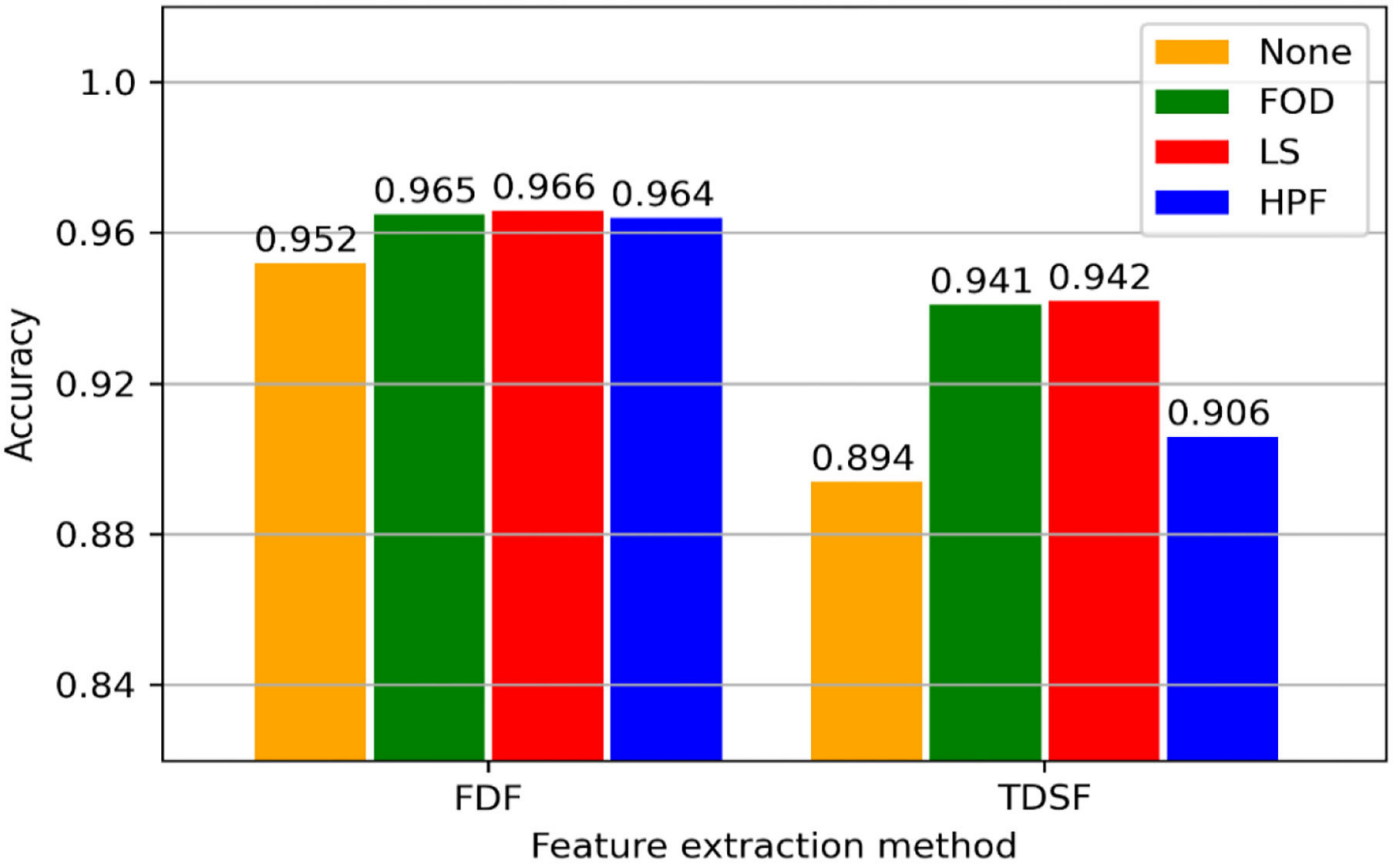
Validation results for each model. “None” indicates the control group in data preprocessing without any data processing. FOD, First-order difference; LS, local slope; HPF, high-pass filter; FDF, frequency-domain features; and TDSF, time-domain statistical features.

The prediction accuracy of machine learning models is shown in Figure 5. The models with frequency-domain features show a higher accuracy in recognition than that with time-domain statistical features. With respect to frequency domain features, three data processing algorithms improve the recognition accuracy of the model, reaching 0.012. In the time-domain feature condition, the local variation extraction algorithm (first-order difference and local slope) has improved by 0.047 compared with the empty operation.

### Comparisons of Machine Learning Algorithms

Multiple supervised classification models are used in this work to compare the performance of k-nearest neighbor (KNN), support vector machine (SVM), decision tree (DT), and XGBoost models on behavior classification. The performance results of various models are presented in Table 5. The highest performance was obtained by combining LS+FDF and XGBoost (accuracy: 0.966 ± 0.010). The model with the lowest performance is the one that uses HPF+TDSF with SVM (accuracy: 0.689 ± 0.091). In the cross-validation, the frequency-domain models are more accurate and robust than the time-domain models. The best performance across all the data processing streams was obtained by XGBoost with an over 89% accuracy.

**Table 5:** The experiment with multi-machine learning (ML) algorithm.

**Data processing**	**Classification algorithm**			
	**KNN**	**SVM**	**DT**	**XGBoost**
FDF	0.900 ± 0.050	0.928 ± 0.038	0.904 ± 0.034	0.952 ± 0.028
FOD + FDF	0.952 ± 0.011	0.964 ± 0.008	0.927 ± 0.019	0.965 ± 0.015
LS + FDF	0.946 ± 0.015	0.961 ± 0.011	0.925 ± 0.016	0.966 ± 0.010
HPF + FDF	0.944 ± 0.015	0.959 ± 0.012	0.919 ± 0.026	0.964 ± 0.016
TDSF	0.792 ± 0.067	0.762 ± 0.110	0.862 ± 0.041	0.894 ± 0.064
FOD + TDSF	0.906 ± 0.028	0.907 ± 0.027	0.918 ± 0.026	0.941 ± 0.035
LS + TDSF	0.903 ± 0.024	0.901 ± 0.027	0.909 ± 0.032	0.942 ± 0.034
HPF + TDSF	0.821 ± 0.062	0.689 ± 0.091	0.883 ± 0.039	0.906 ± 0.060

### Discrimination of Every Behavior

**Figure 6:**
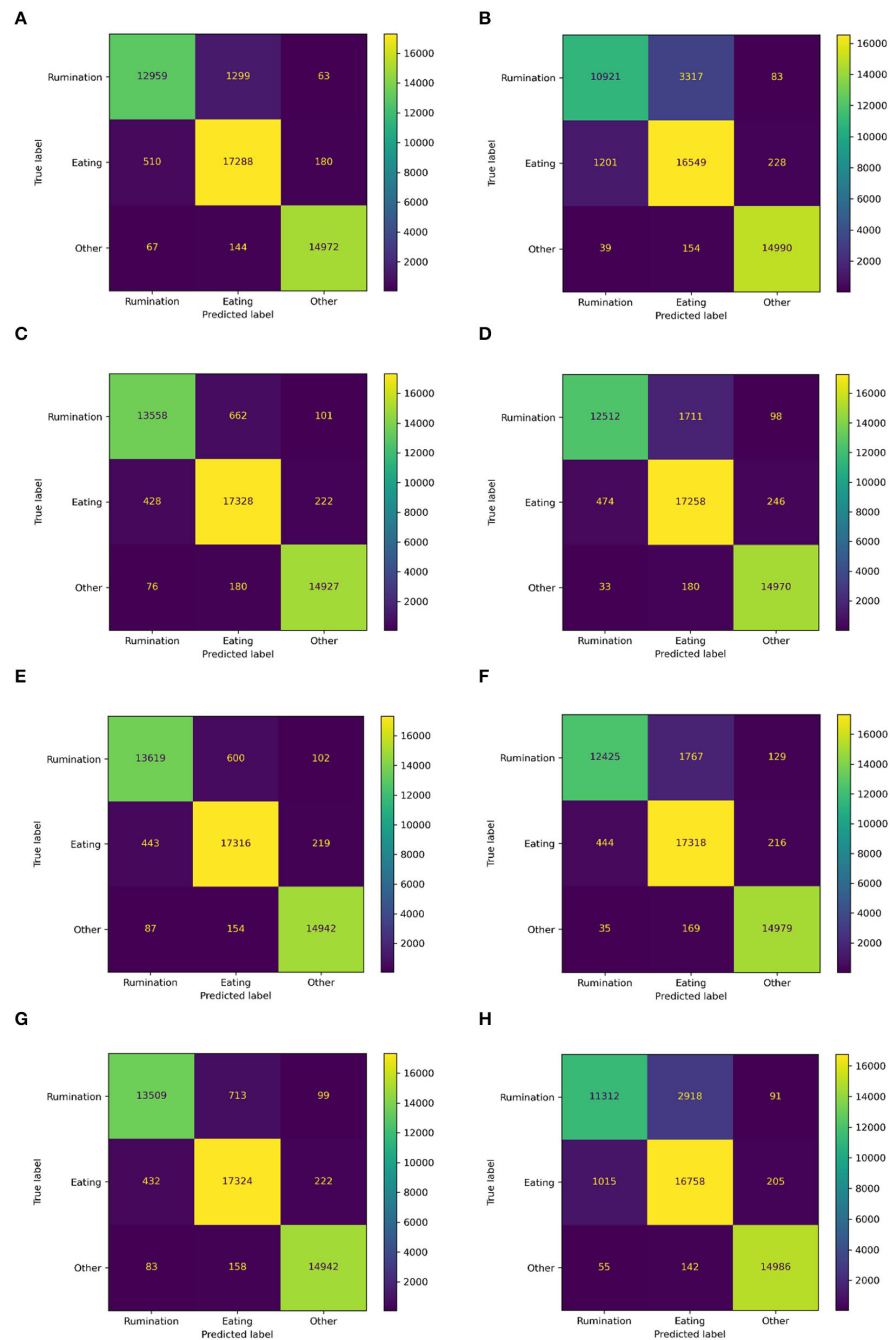
The confusion matrix of each model. FOD, First-order difference; LS, local slope; HPF, high-pass filter; FDF, frequency-domain features; and TDSF, time-domain statistical features. **(A)** FDF, **(B)** TDSF, **(C)** FOD + FDF, **(D)** FOD + TDSF, **(E)** LS + FDF, **(F)** LS + TDSF, **(G)** HPF + FDF, and **(H)** HPF + TDSF.

Figure 6 shows the total confusion matrix for each model and the recognition performance of every behavior is shown in Table 6. The recognition results of rumination and eating behavior were basically consistent with the overall performance. The model achieved the F1 score of 0.96 for rumination and eating recognition by using the local slope or first-order difference and feature extraction using the frequency-domain feature. The worst recognition model is using time-domain features which achieved a 0.82 F1 score for rumination behavior and a 0.87 F1 score for eating behavior.

**Table 6:** Precision, recall, and F1 score of rumination and eating behavior for every model.

**Behavior**	**Model**	**Precision**	**Recall**	**F1 score**
Rumination	FDF	0.96	0.90	0.93
	FOD + FDF	0.96	0.95	0.96
	LS + FDF	0.96	0.95	0.96
	HPF + FDF	0.96	0.94	0.95
	TDSF	0.90	0.76	0.82
	FOD + TDSF	0.96	0.87	0.92
	LS + TDSF	0.96	0.87	0.91
	HPF + TDSF	0.91	0.79	0.85
Eating	FDF	0.92	0.96	0.94
	FOD + FDF	0.95	0.96	0.96
	LS + FDF	0.96	0.96	0.96
	HPF + FDF	0.95	0.96	0.96
	TDSF	0.83	0.92	0.87
	FOD + TDSF	0.90	0.96	0.93
	LS + TDSF	0.90	0.96	0.93
	HPF + TDSF	0.85	0.93	0.89

## Discussion

The jaw movements of cattle are the most obvious features of feeding behavior. The difference between eating and rumination is mainly reflected in the changes in the frequency, amplitude, and trends of jaw movements. Even though the noseband pressure sensor has the advantages in sensing behaviors and strong resistance to non-feeding behaviors, the differences in cattle individuals and resistance to wearing the pressure sensors while wearing the pressure sensors will result in different initial pressure. At this stage, it is unreliable to use the pressure data to process the feeding behavior. Therefore, this article proposed a machine learning approach combined with data preprocessing to provide an effective way for feeding behavior recognition using the pressure sensors in the grazing pasture. The novelty of this article is that the local slope with the combination of the FFT method was used to eliminate the influence of initial pressure, and frequency-domain features were extracted for feeding behavior recognition.

In this article, three data preprocessing methods, namely, first-order difference, high-pass Butterworth filter, and local slope were used to preprocess the raw data with different initial pressure values. Then the frequency-domain and time-domain features were extracted from the preprocessed data and raw data, respectively, and finally, different combination features were applied to the XGB model for behavior classification. Experimental results indict that the accuracy with data preprocessing is significantly higher than that without any preprocessing. Since the initial pressure of the noseband pressure sensor has an impact on the feeding behavior recognition, the influence of the initial pressure was weakened to different extents using the preprocessing methods. The processing of the first-order variance and local slope was reflected in the changing trend of the jaw pressure in the chewing process. During eating and ruminating, chewing speed was different and it can be observed according to the trend of changes in the jaw pressure (45). A high-pass Butterworth filter was used for filtering the low-frequency signal data (lower than 0.3 Hz) which was not the main frequency signal in the process of eating (around 1.1 Hz) and ruminating (around 0.8 Hz). In general, this work is to facilitate the use of the noseband pressure sensor in practice. That is to say, there is no need to consider keeping the same initial pressure while wearing the device. This work will contribute to the promotion of the high-precision chewing behavior perception method.

As for data feature extraction, since the pressure data of feeding behavior shows a strong regularity, the frequency-domain features were better than the time-domain features in feeding behavior recognition. Rumination consists of a series of uniform chewing behaviors that create a regular waveform, while the pattern of eating is irregular. The distribution of the frequency-domain features could present the regularity and provide the XGB classifier with the optimal features. Results indicated that the local slope combined with frequency-domain feature extraction achieved the best performance among three data preprocessing methods, which yielded the accuracy of 0.966, 0.96, and 0.96 on the whole, rumination and eating, respectively.

Concerning the window size, there is no agreed window size to suggest, as it depends on several parameters such as the number of sequences, processing speed, the location of the sensors, and the specific behaviors to detect (6, 23, 46). This article showed that the larger the time window, the higher the identification accuracy, and the longer the execution time took. In the practical scenarios, the raw data will be collected and sent to the server for processing which will bring a lot of power consumption and traffic costs to the wearable detection equipment. A small amount of result data will reduce the data transmission of a single node and in this way, the LORA can be run stably in the low-speed and long-distance communication mode in the future. Therefore, this article balanced the size of the window size and the amount of model calculation and finally chose 10 s as the window size. Although the accuracy is not the highest, it can provide a reference for the embedded feeding behavior recognition model.

During the experiment, the grazing area was relatively empty and the noseband pressure sensor was not damaged during the use. However, if the cattle fight or attack large objects, it is likely to damage the noseband pressure sensor. Therefore, this article will consider using flexible solar cells for power to reduce the power consumption of the whole equipment, which can reduce the volume of the battery in the noseband pressure equipment. Moreover, the outer shell of the equipment will be made of harder material to withstand greater impact force and meanwhile, the hard shell will be covered with a flexible shell to avoid damage to cattle.

## Conclusions

Automatic monitoring of feeding behavior especially rumination and eating in cattle is of importance to keep track of animals' health and growth conditions and disease warnings. In this article, the noseband pressure sensor was used to collect the behavior data of cattle, however, it was difficult to achieve the high-precision accuracy of feeding behavior recognition by using the existing algorithms with raw data. For future potential applications, three data preprocessing methods and two feature extraction algorithms were evaluated in this study. It is concluded that the XGB classification model in combination with the local slope and frequency-domain feature achieved the F1 score of 0.96 and accuracy of 0.966 for feeding behavior recognition. The proposed approach is suitable for processing pressure data with a wide range of variations, which can avoid the adjustment of the pressure sensor while wearing the device. This work will help reduce labor consumption and contribute to the standardized application and promotion of the noseband pressure sensors.

Since the difference in breeds and ages of cattle will potentially influence the model performance, future work in this study will extend to more kinds and ages of cows, to have better scalability and ensure that the proposed method applies to all kinds of farms.

## Data Availability Statement

The raw data supporting the conclusions of this article will be made available by the authors, without undue reservation.

## Ethics Statement

The animal study was reviewed and approved by Jiangxi Academy of Agricultural Sciences, Institute of Animal Husbandry and Veterinary Laboratory, and Animal Ethics Committee. Written informed consent was obtained from the owners for the participation of their animals in this study.

## Author Contributions

GC and BX: conceptualization, methodology, software, formal analysis, writing, original–draft preparation, and visualization. GC, BX, and CL: validation. CL and YG: investigation and data curation. CL, YG, and ZC: resources, supervision, and project administration. GC, CL, and HS: writing–review and editing. All the authors contributed to the article and approved the submitted version.

## Funding

This research was funded by the Youth Science Foundation Project of Jiangxi Province (20192ACBL21023), Jiangxi Province Research Collaborative Innovation Special Project for Modern Agriculture (JXXTCXNLTS202106), Jiangxi Academy of Agriculture Sciences Innovation Fund for Doctor Launches Project (20181CBS006), National Natural Science Foundation of China, China (32060776), and Jiangxi Province 03 Special Project and 5G Project (20204ABC03A09).

## Conflict of Interest

The authors declare that the research was conducted in the absence of any commercial or financial relationships that could be construed as a potential conflict of interest.

## Publisher's Note

All claims expressed in this article are solely those of the authors and do not necessarily represent those of their affiliated organizations, or those of the publisher, the editors and the reviewers. Any product that may be evaluated in this article, or claim that may be made by its manufacturer, is not guaranteed or endorsed by the publisher.
